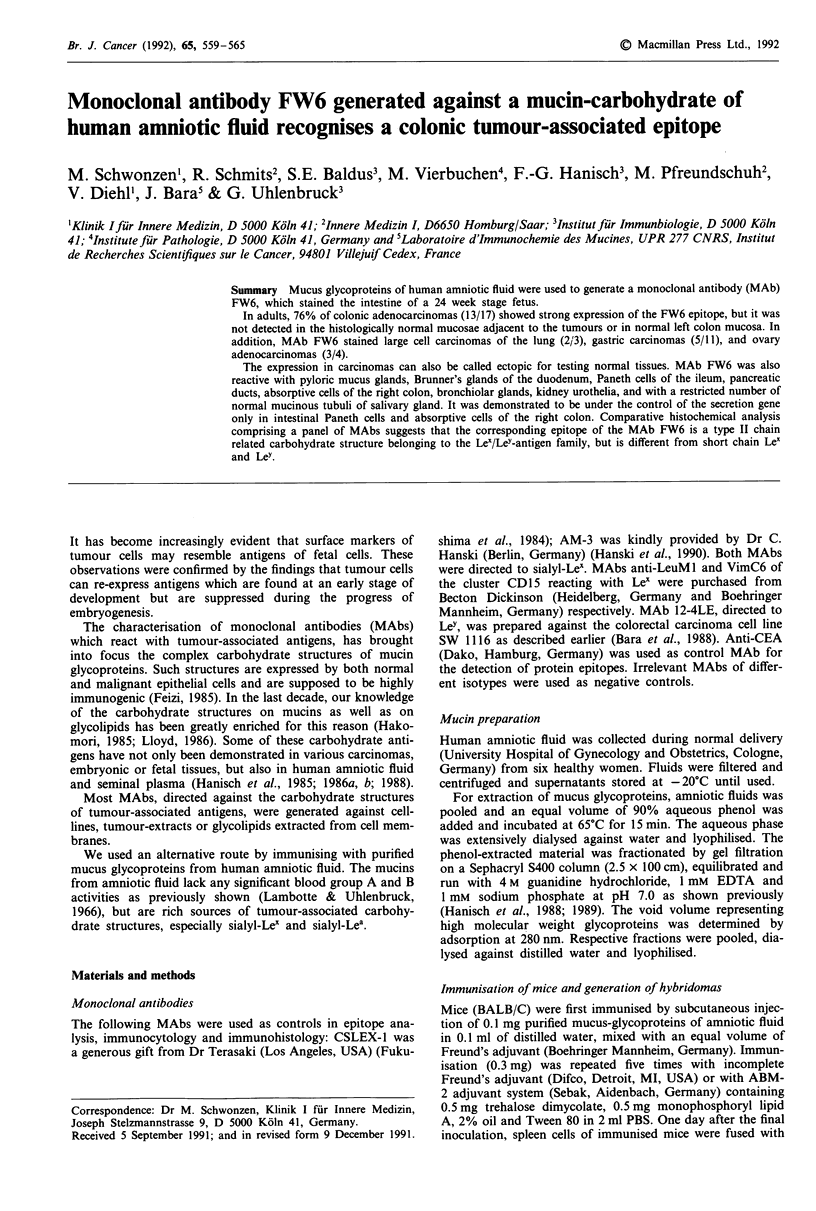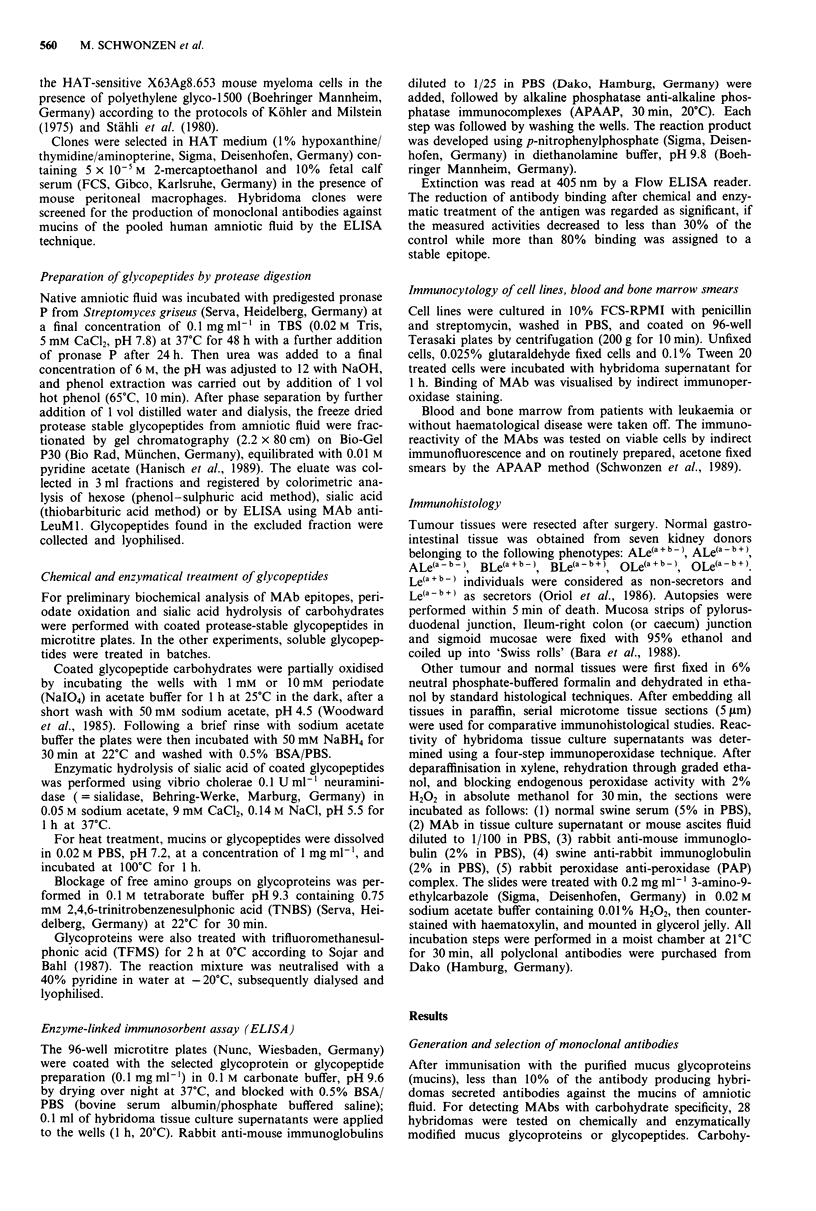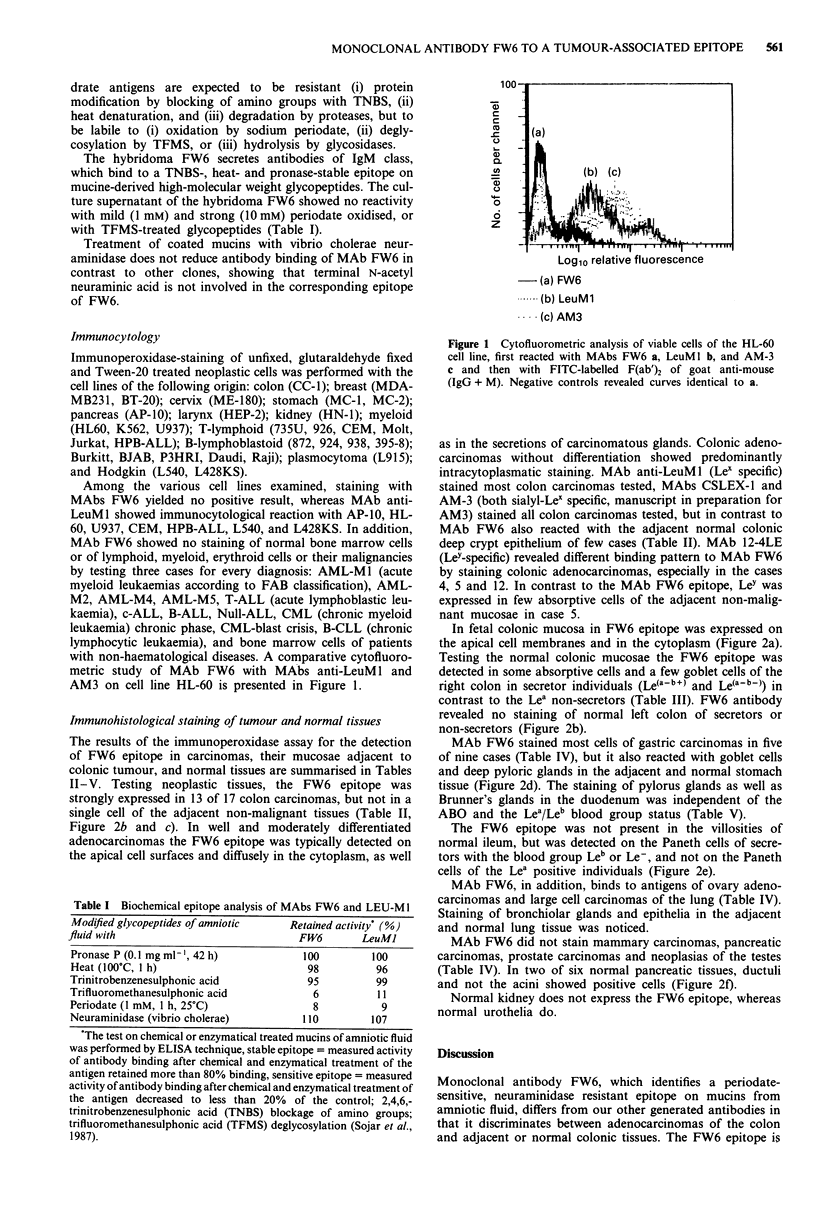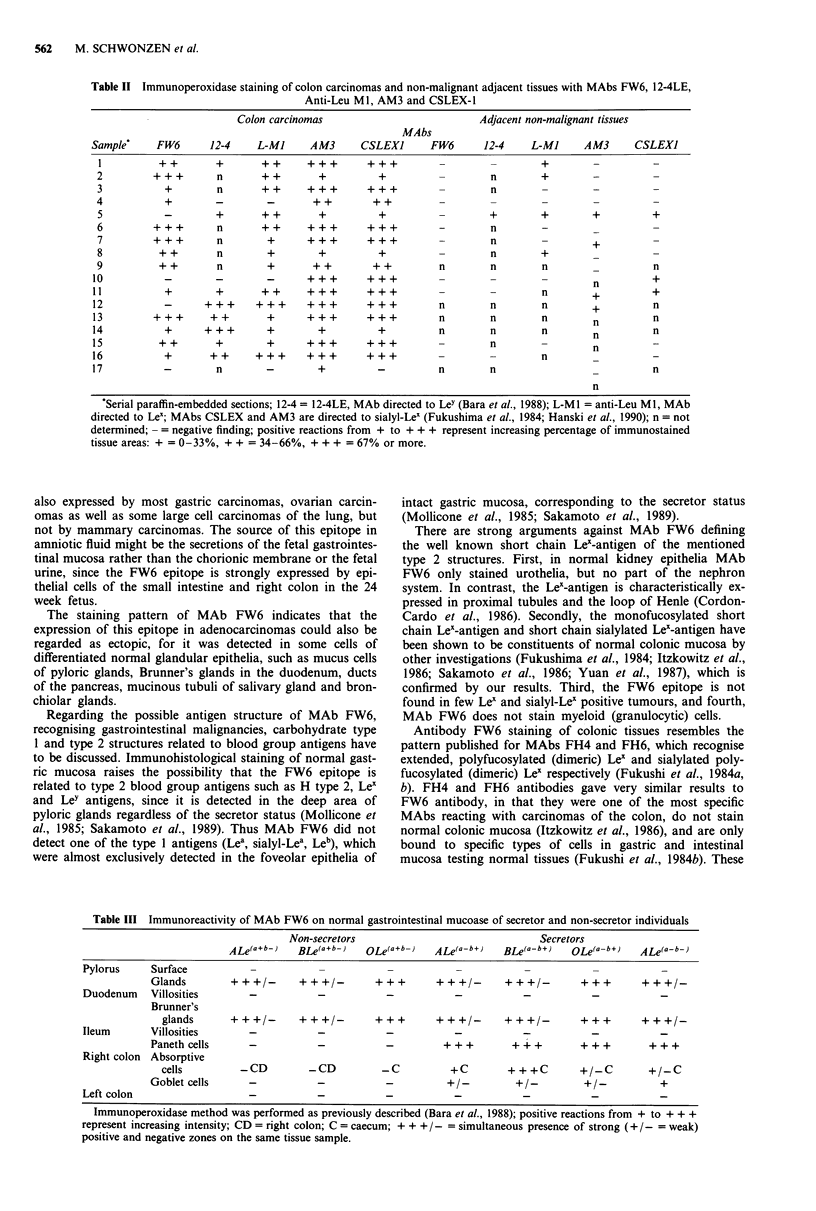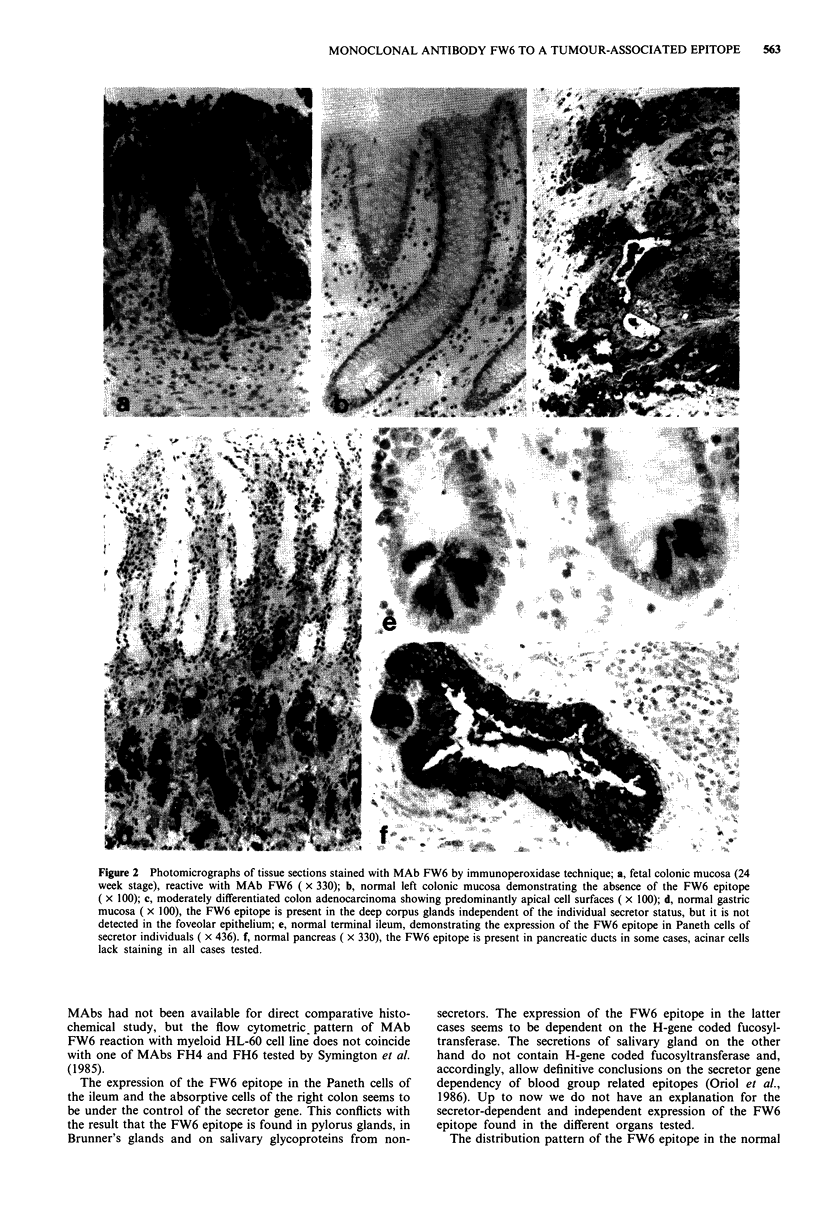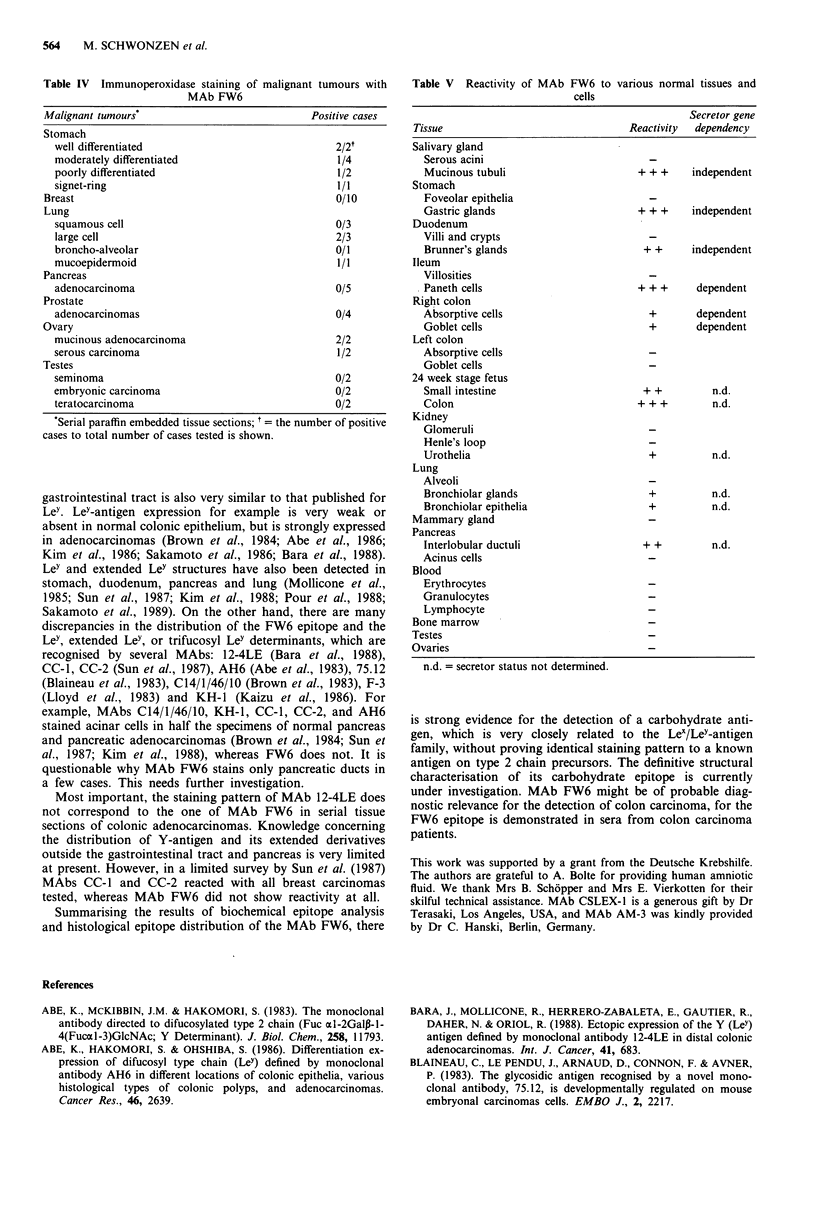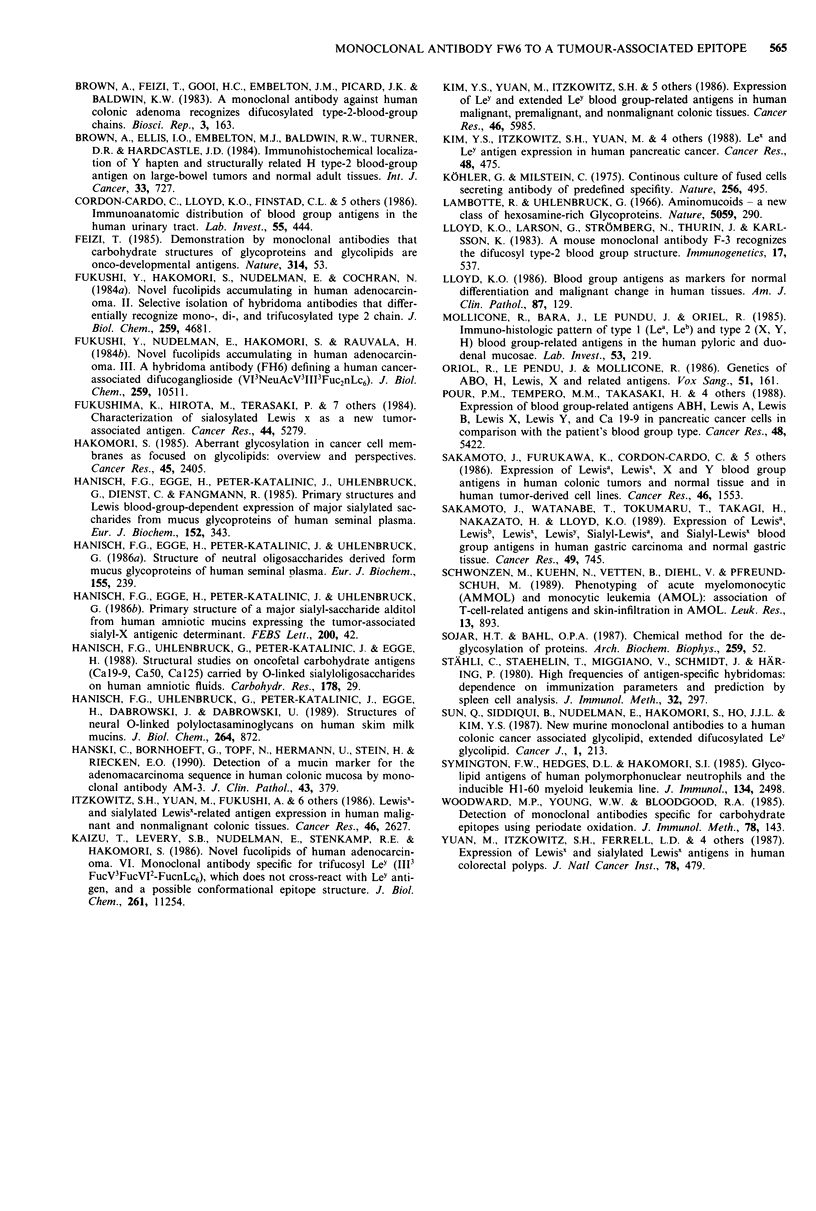# Monoclonal antibody FW6 generated against a mucin-carbohydrate of human amniotic fluid recognises a colonic tumour-associated epitope.

**DOI:** 10.1038/bjc.1992.114

**Published:** 1992-04

**Authors:** M. Schwonzen, R. Schmits, S. E. Baldus, M. Vierbuchen, F. G. Hanisch, M. Pfreundschuh, V. Diehl, J. Bara, G. Uhlenbruck

**Affiliations:** Klinik I für Innere Medizin, Köln, Germany.

## Abstract

**Images:**


Br. J. Cancer (1992), 65, 559-565 © Macmillan Press Ltd., 1992

### Monoclonal antibody FW6 generated against a mucin-carbohydrate of human amniotic fluid recognises a colonic tumour-associated epitope

M. Schwonzen[1], R. Schmits[2], S.E. Baldus[3], M. Vierbuchen[4], F.-G. Hanisch[3], M. Pfreundschuh[2], V. Diehl[1], J. Bara[5] & G. Uhlenbruck[3]

[1]*Klinik I für Innere Medizin, D 5000 Köln 41;* [2]*Innere Medizin I, D6650 Homburg/Saar;* [3]*Institut für Immunbiologie, D 5000 Köln 41;* [4]*Institute für Pathologie, D 5000 Köln 41, Germany and* [5]*Laboratoire d'Immunochemie des Mucines, UPR 277 CNRS, Institut de Recherches Scientifiques sur le Cancer, 94801 Villejuif Cedex, France*

**Summary** Mucus glycoproteins of human amniotic fluid were used to generate a monoclonal antibody (MAb) FW6, which stained the intestine of a 24 week stage fetus.

In adults, 76% of colonic adenocarcinomas (13/17) showed strong expression of the FW6 epitope, but it was not detected in the histologically normal mucosae adjacent to the tumours or in normal left colon mucosa. In addition, MAb FW6 stained large cell carcinomas of the lung (2/3), gastric carcinomas (5/11), and ovary adenocarcinomas (3/4).

The expression in carcinomas can also be called ectopic for testing normal tissues. MAb FW6 was also reactive with pyloric mucus glands, Brunner's glands of the duodenum, Paneth cells of the ileum, pancreatic ducts, absorptive cells of the right colon, bronchiolar glands, kidney urothelia, and with a restricted number of normal mucinous tubuli of salivary gland. It was demonstrated to be under the control of the secretion gene only in intestinal Paneth cells and absorptive cells of the right colon. Comparative histochemical analysis comprising a panel of MAbs suggests that the corresponding epitope of the MAb FW6 is a type II chain related carbohydrate structure belonging to the $Le^x$/$Le^y$-antigen family, but is different from short chain $Le^x$ and $Le^y$.

It has become increasingly evident that surface markers of tumour cells may resemble antigens of fetal cells. These observations were confirmed by the findings that tumour cells can re-express antigens which are found at an early stage of development but are suppressed during the progress of embryogenesis.

The characterisation of monoclonal antibodies (MAbs) which react with tumour-associated antigens, has brought into focus the complex carbohydrate structures of mucin glycoproteins. Such structures are expressed by both normal and malignant epithelial cells and are supposed to be highly immunogenic (Feizi, 1985). In the last decade, our knowledge of the carbohydrate structures on mucins as well as on glycolipids has been greatly enriched for this reason (Hakomori, 1985; Lloyd, 1986). Some of these carbohydrate antigens have not only been demonstrated in various carcinomas, embryonic or fetal tissues, but also in human amniotic fluid and seminal plasma (Hanisch *et al.*, 1985; 1986*a*, *b*; 1988).

Most MAbs, directed against the carbohydrate structures of tumour-associated antigens, were generated against cell-lines, tumour-extracts or glycolipids extracted from cell membranes.

We used an alternative route by immunising with purified mucus glycoproteins from human amniotic fluid. The mucins from amniotic fluid lack any significant blood group A and B activities as previously shown (Lambotte & Uhlenbruck, 1966), but are rich sources of tumour-associated carbohydrate structures, especially sialyl-$Le^x$ and sialyl-$Le^a$.

### Materials and methods

### *Monoclonal antibodies*

The following MAbs were used as controls in epitope analysis, immunocytology and immunohistology: CSLEX-1 was a generous gift from Dr Terasaki (Los Angeles, USA) (Fukushima *et al.*, 1984); AM-3 was kindly provided by Dr C. Hanski (Berlin, Germany) (Hanski *et al.*, 1990). Both MAbs were directed to sialyl-$Le^x$. MAbs anti-LeuM1 and VimC6 of the cluster CD15 reacting with $Le^x$ were purchased from Becton Dickinson (Heidelberg, Germany and Boehringer Mannheim, Germany) respectively. MAb 12-4LE, directed to $Le^y$, was prepared against the colorectal carcinoma cell line SW 1116 as described earlier (Bara *et al.*, 1988). Anti-CEA (Dako, Hamburg, Germany) was used as control MAb for the detection of protein epitopes. Irrelevant MAbs of different isotypes were used as negative controls.

### *Mucin preparation*

Human amniotic fluid was collected during normal delivery (University Hospital of Gynecology and Obstetrics, Cologne, Germany) from six healthy women. Fluids were filtered and centrifuged and supernatants stored at $-20°C$ until used.

For extraction of mucus glycoproteins, amniotic fluids was pooled and an equal volume of 90% aqueous phenol was added and incubated at 65°C for 15 min. The aqueous phase was extensively dialysed against water and lyophilised. The phenol-extracted material was fractionated by gel filtration on a Sephacryl S400 column (2.5 × 100 cm), equilibrated and run with 4 M guanidine hydrochloride, 1 mM EDTA and 1 mM sodium phosphate at pH 7.0 as shown previously (Hanisch *et al.*, 1988; 1989). The void volume representing high molecular weight glycoproteins was determined by adsorption at 280 nm. Respective fractions were pooled, dialysed against distilled water and lyophilised.

### *Immunisation of mice and generation of hybridomas*

Mice (BALB/C) were first immunised by subcutaneous injection of 0.1 mg purified mucus-glycoproteins of amniotic fluid in 0.1 ml of distilled water, mixed with an equal volume of Freund's adjuvant (Boehringer Mannheim, Germany). Immunisation (0.3 mg) was repeated five times with incomplete Freund's adjuvant (Difco, Detroit, MI, USA) or with ABM-2 adjuvant system (Sebak, Aidenbach, Germany) containing 0.5 mg trehalose dimycolate, 0.5 mg monophosphoryl lipid A, 2% oil and Tween 80 in 2 ml PBS. One day after the final inoculation, spleen cells of immunised mice were fused with

Correspondence: Dr M. Schwonzen, Klinik I für Innere Medizin, Joseph Stelzmannstrasse 9, D 5000 Köln 41, Germany.
Received 5 September 1991; and in revised form 9 December 1991.

the HAT-sensitive X63Ag8.653 mouse myeloma cells in the presence of polyethylene glyco-1500 (Boehringer Mannheim, Germany) according to the protocols of Köhler and Milstein (1975) and Stähli *et al.* (1980).

Clones were selected in HAT medium (1% hypoxanthine/thymidine/aminopterine, Sigma, Deisenhofen, Germany) containing $5 \times 10^{-5}$ M 2-mercaptoethanol and 10% fetal calf serum (FCS, Gibco, Karlsruhe, Germany) in the presence of mouse peritoneal macrophages. Hybridoma clones were screened for the production of monoclonal antibodies against mucins of the pooled human amniotic fluid by the ELISA technique.

*Preparation of glycopeptides by protease digestion*

Native amniotic fluid was incubated with predigested pronase P from *Streptomyces griseus* (Serva, Heidelberg, Germany) at a final concentration of 0.1 mg ml$^{-1}$ in TBS (0.02 M Tris, 5 mM $CaCl_2$, pH 7.8) at 37°C for 48 h with a further addition of pronase P after 24 h. Then urea was added to a final concentration of 6 M, the pH was adjusted to 12 with NaOH, and phenol extraction was carried out by addition of 1 vol hot phenol (65°C, 10 min). After phase separation by further addition of 1 vol distilled water and dialysis, the freeze dried protease stable glycopeptides from amniotic fluid were fractionated by gel chromatography (2.2 × 80 cm) on Bio-Gel P30 (Bio Rad, München, Germany), equilibrated with 0.01 M pyridine acetate (Hanisch *et al.*, 1989). The eluate was collected in 3 ml fractions and registered by colorimetric analysis of hexose (phenol–sulphuric acid method), sialic acid (thiobarbituric acid method) or by ELISA using MAb anti-LeuM1. Glycopeptides found in the excluded fraction were collected and lyophilised.

*Chemical and enzymatical treatment of glycopeptides*

For preliminary biochemical analysis of MAb epitopes, periodate oxidation and sialic acid hydrolysis of carbohydrates were performed with coated protease-stable glycopeptides in microtitre plates. In the other experiments, soluble glycopeptides were treated in batches.

Coated glycopeptide carbohydrates were partially oxidised by incubating the wells with 1 mM or 10 mM periodate ($NaIO_4$) in acetate buffer for 1 h at 25°C in the dark, after a short wash with 50 mM sodium acetate, pH 4.5 (Woodward *et al.*, 1985). Following a brief rinse with sodium acetate buffer the plates were then incubated with 50 mM $NaBH_4$ for 30 min at 22°C and washed with 0.5% BSA/PBS.

Enzymatic hydrolysis of sialic acid of coated glycopeptides was performed using vibrio cholerae 0.1 U ml$^{-1}$ neuraminidase ( = sialidase, Behring-Werke, Marburg, Germany) in 0.05 M sodium acetate, 9 mM $CaCl_2$, 0.14 M NaCl, pH 5.5 for 1 h at 37°C.

For heat treatment, mucins or glycopeptides were dissolved in 0.02 M PBS, pH 7.2, at a concentration of 1 mg ml$^{-1}$, and incubated at 100°C for 1 h.

Blockage of free amino groups on glycoproteins was performed in 0.1 M tetraborate buffer pH 9.3 containing 0.75 mM 2,4,6-trinitrobenzenesulphonic acid (TNBS) (Serva, Heidelberg, Germany) at 22°C for 30 min.

Glycoproteins were also treated with trifluoromethanesulphonic acid (TFMS) for 2 h at 0°C according to Sojar and Bahl (1987). The reaction mixture was neutralised with a 40% pyridine in water at −20°C, subsequently dialysed and lyophilised.

*Enzyme-linked immunosorbent assay (ELISA)*

The 96-well microtitre plates (Nunc, Wiesbaden, Germany) were coated with the selected glycoprotein or glycopeptide preparation (0.1 mg ml$^{-1}$) in 0.1 M carbonate buffer, pH 9.6 by drying over night at 37°C, and blocked with 0.5% BSA/PBS (bovine serum albumin/phosphate buffered saline); 0.1 ml of hybridoma tissue culture supernatants were applied to the wells (1 h, 20°C). Rabbit anti-mouse immunoglobulins diluted to 1/25 in PBS (Dako, Hamburg, Germany) were added, followed by alkaline phosphatase anti-alkaline phosphatase immunocomplexes (APAAP, 30 min, 20°C). Each step was followed by washing the wells. The reaction product was developed using *p*-nitrophenylphosphate (Sigma, Deisenhofen, Germany) in diethanolamine buffer, pH 9.8 (Boehringer Mannheim, Germany).

Extinction was read at 405 nm by a Flow ELISA reader. The reduction of antibody binding after chemical and enzymatic treatment of the antigen was regarded as significant, if the measured activities decreased to less than 30% of the control while more than 80% binding was assigned to a stable epitope.

*Immunocytology of cell lines, blood and bone marrow smears*

Cell lines were cultured in 10% FCS-RPMI with penicillin and streptomycin, washed in PBS, and coated on 96-well Terasaki plates by centrifugation (200 g for 10 min). Unfixed cells, 0.025% glutaraldehyde fixed cells and 0.1% Tween 20 treated cells were incubated with hybridoma supernatant for 1 h. Binding of MAb was visualised by indirect immunoperoxidase staining.

Blood and bone marrow from patients with leukaemia or without haematological disease were taken off. The immunoreactivity of the MAbs was tested on viable cells by indirect immunofluorescence and on routinely prepared, acetone fixed smears by the APAAP method (Schwonzen *et al.*, 1989).

*Immunohistology*

Tumour tissues were resected after surgery. Normal gastrointestinal tissue was obtained from seven kidney donors belonging to the following phenotypes: $ALe^{(a+b-)}$, $ALe^{(a-b+)}$, $ALe^{(a-b-)}$, $BLe^{(a+b-)}$, $BLe^{(a-b+)}$, $OLe^{(a+b-)}$, $OLe^{(a-b+)}$. $Le^{(a+b-)}$ individuals were considered as non-secretors and $Le^{(a-b+)}$ as secretors (Oriol *et al.*, 1986). Autopsies were performed within 5 min of death. Mucosa strips of pylorus-duodenal junction, Ileum-right colon (or caecum) junction and sigmoid mucosae were fixed with 95% ethanol and coiled up into 'Swiss rolls' (Bara *et al.*, 1988).

Other tumour and normal tissues were first fixed in 6% neutral phosphate-buffered formalin and dehydrated in ethanol by standard histological techniques. After embedding all tissues in paraffin, serial microtome tissue sections (5 μm) were used for comparative immunohistological studies. Reactivity of hybridoma tissue culture supernatants was determined using a four-step immunoperoxidase technique. After deparaffinisation in xylene, rehydration through graded ethanol, and blocking endogenous peroxidase activity with 2% $H_2O_2$ in absolute methanol for 30 min, the sections were incubated as follows: (1) normal swine serum (5% in PBS), (2) MAb in tissue culture supernatant or mouse ascites fluid diluted to 1/100 in PBS, (3) rabbit anti-mouse immunoglobulin (2% in PBS), (4) swine anti-rabbit immunoglobulin (2% in PBS), (5) rabbit peroxidase anti-peroxidase (PAP) complex. The slides were treated with 0.2 mg ml$^{-1}$ 3-amino-9-ethylcarbazole (Sigma, Deisenhofen, Germany) in 0.02 M sodium acetate buffer containing 0.01% $H_2O_2$, then counterstained with haematoxylin, and mounted in glycerol jelly. All incubation steps were performed in a moist chamber at 21°C for 30 min, all polyclonal antibodies were purchased from Dako (Hamburg, Germany).

### Results

*Generation and selection of monoclonal antibodies*

After immunisation with the purified mucus glycoproteins (mucins), less than 10% of the antibody producing hybridomas secreted antibodies against the mucins of amniotic fluid. For detecting MAbs with carbohydrate specificity, 28 hybridomas were tested on chemically and enzymatically modified mucus glycoproteins or glycopeptides. Carbohydrate antigens are expected to be resistant (i) protein modification by blocking of amino groups with TNBS, (ii) heat denaturation, and (iii) degradation by proteases, but to be labile to (i) oxidation by sodium periodate, (ii) deglycosylation by TFMS, or (iii) hydrolysis by glycosidases.

The hybridoma FW6 secretes antibodies of IgM class, which bind to a TNBS-, heat- and pronase-stable epitope on mucine-derived high-molecular weight glycopeptides. The culture supernatant of the hybridoma FW6 showed no reactivity with mild (1 mM) and strong (10 mM) periodate oxidised, or with TFMS-treated glycopeptides (Table I).

Treatment of coated mucins with vibrio cholerae neuraminidase does not reduce antibody binding of MAb FW6 in contrast to other clones, showing that terminal N-acetyl neuraminic acid is not involved in the corresponding epitope of FW6.

### *Immunocytology*

Immunoperoxidase-staining of unfixed, glutaraldehyde fixed and Tween-20 treated neoplastic cells was performed with the cell lines of the following origin: colon (CC-1); breast (MDA-MB231, BT-20); cervix (ME-180); stomach (MC-1, MC-2); pancreas (AP-10); larynx (HEP-2); kidney (HN-1); myeloid (HL60, K562, U937); T-lymphoid (735U, 926, CEM, Molt, Jurkat, HPB-ALL); B-lymphoblastoid (872, 924, 938, 395-8); Burkitt, BJAB, P3HRI, Daudi, Raji); plasmocytoma (L915); and Hodgkin (L540, L428KS).

Among the various cell lines examined, staining with MAbs FW6 yielded no positive result, whereas MAb anti-LeuM1 showed immunocytological reaction with AP-10, HL-60, U937, CEM, HPB-ALL, L540, and L428KS. In addition, MAb FW6 showed no staining of normal bone marrow cells or of lymphoid, myeloid, erythroid cells or their malignancies by testing three cases for every diagnosis: AML-M1 (acute myeloid leukaemias according to FAB classification), AML-M2, AML-M4, AML-M5, T-ALL (acute lymphoblastic leukaemia), c-ALL, B-ALL, Null-ALL, CML (chronic myeloid leukaemia) chronic phase, CML-blast crisis, B-CLL (chronic lymphocytic leukaemia), and bone marrow cells of patients with non-haematological diseases. A comparative cytofluorometric study of MAb FW6 with MAbs anti-LeuM1 and AM3 on cell line HL-60 is presented in Figure 1.

### *Immunohistological staining of tumour and normal tissues*

The results of the immunoperoxidase assay for the detection of FW6 epitope in carcinomas, their mucosae adjacent to colonic tumour, and normal tissues are summarised in Tables II–V. Testing neoplastic tissues, the FW6 epitope was strongly expressed in 13 of 17 colon carcinomas, but not in a single cell of the adjacent non-malignant tissues (Table II, Figure 2*b* and *c*). In well and moderately differentiated adenocarcinomas the FW6 epitope was typically detected on the apical cell surfaces and diffusely in the cytoplasm, as well

**Table I** Biochemical epitope analysis of MAbs FW6 and LEU-M1

| *Modified glycopeptides of amniotic fluid with* | *Retained activity* (%)* FW6 | *LeuM1* |
|---|---|---|
| Pronase P (0.1 mg ml$^{-1}$, 42 h) | 100 | 100 |
| Heat (100°C, 1 h) | 98 | 96 |
| Trinitrobenzenesulphonic acid | 95 | 99 |
| Trifluoromethanesulphonic acid | 6 | 11 |
| Periodate (1 mM, 1 h, 25°C) | 8 | 9 |
| Neuraminidase (vibrio cholerae) | 110 | 107 |

*The test on chemical or enzymatical treated mucins of amniotic fluid was performed by ELISA technique, stable epitope = measured activity of antibody binding after chemical and enzymatical treatment of the antigen retained more than 80% binding, sensitive epitope = measured activity of antibody binding after chemical and enzymatical treatment of the antigen decreased to less than 20% of the control; 2,4,6,-trinitrobenzenesulphonic acid (TNBS) blockage of amino groups; trifluoromethanesulphonic acid (TFMS) deglycosylation (Sojar *et al.*, 1987).

**Figure 1** Cytofluorometric analysis of viable cells of the HL-60 cell line, first reacted with MAbs FW6 **a**, LeuM1 **b**, and AM-3 **c** and then with FITC-labelled F(ab')$_2$ of goat anti-mouse (IgG + M). Negative controls revealed curves identical to **a**.

as in the secretions of carcinomatous glands. Colonic adenocarcinomas without differentiation showed predominantly intracytoplasmatic staining. MAb anti-LeuM1 (Le$^x$ specific) stained most colon carcinomas tested, MAbs CSLEX-1 and AM-3 (both sialyl-Le$^x$ specific, manuscript in preparation for AM3) stained all colon carcinomas tested, but in contrast to MAb FW6 also reacted with the adjacent normal colonic deep crypt epithelium of few cases (Table II). MAb 12-4LE (Le$^y$-specific) revealed different binding pattern to MAb FW6 by staining colonic adenocarcinomas, especially in the cases 4, 5 and 12. In contrast to the MAb FW6 epitope, Le$^y$ was expressed in few absorptive cells of the adjacent non-malignant mucosae in case 5.

In fetal colonic mucosa in FW6 epitope was expressed on the apical cell membranes and in the cytoplasm (Figure 2a). Testing the normal colonic mucosae the FW6 epitope was detected in some absorptive cells and a few goblet cells of the right colon in secretor individuals (Le$^{(a-b+)}$ and Le$^{(a-b-)}$) in contrast to the Le$^a$ non-secretors (Table III). FW6 antibody revealed no staining of normal left colon of secretors or non-secretors (Figure 2b).

MAb FW6 stained most cells of gastric carcinomas in five of nine cases (Table IV), but it also reacted with goblet cells and deep pyloric glands in the adjacent and normal stomach tissue (Figure 2d). The staining of pylorus glands as well as Brunner's glands in the duodenum was independent of the ABO and the Le$^a$/Le$^b$ blood group status (Table V).

The FW6 epitope was not present in the villosities of normal ileum, but was detected on the Paneth cells of secretors with the blood group Le$^b$ or Le$^-$, and not on the Paneth cells of the Le$^a$ positive individuals (Figure 2e).

MAb FW6, in addition, binds to antigens of ovary adenocarcinomas and large cell carcinomas of the lung (Table IV). Staining of bronchiolar glands and epithelia in the adjacent and normal lung tissue was noticed.

MAb FW6 did not stain mammary carcinomas, pancreatic carcinomas, prostate carcinomas and neoplasias of the testes (Table IV). In two of six normal pancreatic tissues, ductuli and not the acini showed positive cells (Figure 2f).

Normal kidney does not express the FW6 epitope, whereas normal urothelia do.

### Discussion

Monoclonal antibody FW6, which identifies a periodate-sensitive, neuraminidase resistant epitope on mucins from amniotic fluid, differs from our other generated antibodies in that it discriminates between adenocarcinomas of the colon and adjacent or normal colonic tissues. The FW6 epitope is

**Table II** Immunoperoxidase staining of colon carcinomas and non-malignant adjacent tissues with MAbs FW6, 12-4LE, Anti-Leu M1, AM3 and CSLEX-1

| | *Colon carcinomas* | | | | | *MAbs* *Adjacent non-malignant tissues* | | | | |
|---|---|---|---|---|---|---|---|---|---|---|
| *Sample** | *FW6* | *12-4* | *L-M1* | *AM3* | *CSLEX1* | *FW6* | *12-4* | *L-M1* | *AM3* | *CSLEX1* |
| 1 | + + | + | + + | + + + | + + + | – | – | + | – | – |
| 2 | + + + | n | + + | + | + | – | n | + | – | – |
| 3 | + | n | + + | + + + | + + + | – | n | – | – | – |
| 4 | + | – | – | + + | + + | – | – | – | – | – |
| 5 | – | + | + + | + | + | – | + | + | + | + |
| 6 | + + + | n | + + | + + + | + + + | – | n | – | – | – |
| 7 | + + + | n | + | + + + | + + + | – | n | – | + | – |
| 8 | + + | n | + | + | + | – | n | + | – | – |
| 9 | + + | n | + | + + | + + | n | n | n | – | n |
| 10 | – | – | – | + + + | + + + | – | – | – | n | + |
| 11 | + | + | + + | + + + | + + + | – | – | n | + | + |
| 12 | – | + + + | + + + | + + + | + + + | n | n | n | + | n |
| 13 | + + + | + + | + | + + + | + + + | n | n | n | n | n |
| 14 | + | + + + | + | + | + | n | n | n | n | n |
| 15 | + + | + | + | + + + | + + + | – | n | – | n | – |
| 16 | + | + + | + + + | + + + | + + + | – | – | n | – | – |
| 17 | – | n | – | + | – | n | n | | – | n |
| | | | | | | | | | n | |

*Serial paraffin-embedded sections; 12-4 = 12-4LE, MAb directed to $Le^y$ (Bara *et al.*, 1988); L-M1 = anti-Leu M1, MAb directed to $Le^x$; MAbs CSLEX and AM3 are directed to sialyl-$Le^x$ (Fukushima *et al.*, 1984; Hanski *et al.*, 1990); n = not determined; – = negative finding; positive reactions from + to + + + represent increasing percentage of immunostained tissue areas: + = 0–33%, + + = 34–66%, + + + = 67% or more.

also expressed by most gastric carcinomas, ovarian carcinomas as well as some large cell carcinomas of the lung, but not by mammary carcinomas. The source of this epitope in amniotic fluid might be the secretions of the fetal gastrointestinal mucosa rather than the chorionic membrane or the fetal urine, since the FW6 epitope is strongly expressed by epithelial cells of the small intestine and right colon in the 24 week fetus.

The staining pattern of MAb FW6 indicates that the expression of this epitope in adenocarcinomas could also be regarded as ectopic, for it was detected in some cells of differentiated normal glandular epithelia, such as mucus cells of pyloric glands, Brunner's glands in the duodenum, ducts of the pancreas, mucinous tubuli of salivary gland and bronchiolar glands.

Regarding the possible antigen structure of MAb FW6, recognising gastrointestinal malignancies, carbohydrate type 1 and type 2 structures related to blood group antigens have to be discussed. Immunohistological staining of normal gastric mucosa raises the possibility that the FW6 epitope is related to type 2 blood group antigens such as H type 2, $Le^x$ and $Le^y$ antigens, since it is detected in the deep area of pyloric glands regardless of the secretor status (Mollicone *et al.*, 1985; Sakamoto *et al.*, 1989). Thus MAb FW6 did not detect one of the type 1 antigens ($Le^a$, sialyl-$Le^a$, $Le^b$), which were almost exclusively detected in the foveolar epithelia of intact gastric mucosa, corresponding to the secretor status (Mollicone *et al.*, 1985; Sakamoto *et al.*, 1989).

There are strong arguments against MAb FW6 defining the well known short chain $Le^x$-antigen of the mentioned type 2 structures. First, in normal kidney epithelia MAb FW6 only stained urothelia, but no part of the nephron system. In contrast, the $Le^x$-antigen is characteristically expressed in proximal tubules and the loop of Henle (Cordon-Cardo *et al.*, 1986). Secondly, the monofucosylated short chain $Le^x$-antigen and short chain sialylated $Le^x$-antigen have been shown to be constituents of normal colonic mucosa by other investigations (Fukushima *et al.*, 1984; Itzkowitz *et al.*, 1986; Sakamoto *et al.*, 1986; Yuan *et al.*, 1987), which is confirmed by our results. Third, the FW6 epitope is not found in few $Le^x$ and sialyl-$Le^x$ positive tumours, and fourth, MAb FW6 does not stain myeloid (granulocytic) cells.

Antibody FW6 staining of colonic tissues resembles the pattern published for MAbs FH4 and FH6, which recognise extended, polyfucosylated (dimeric) $Le^x$ and sialylated polyfucosylated (dimeric) $Le^x$ respectively (Fukushi *et al.*, 1984*a*, *b*). FH4 and FH6 antibodies gave very similar results to FW6 antibody, in that they were one of the most specific MAbs reacting with carcinomas of the colon, do not stain normal colonic mucosa (Itzkowitz *et al.*, 1986), and are only bound to specific types of cells in gastric and intestinal mucosa testing normal tissues (Fukushi *et al.*, 1984*b*). These

**Table III** Immunoreactivity of MAb FW6 on normal gastrointestinal mucoase of secretor and non-secretor individuals

| | | *Non-secretors* | | | *Secretors* | | | |
|---|---|---|---|---|---|---|---|---|
| | | $ALe^{(a+b-)}$ | $BLe^{(a+b-)}$ | $OLe^{(a+b-)}$ | $ALe^{(a-b+)}$ | $BLe^{(a-b+)}$ | $OLe^{(a-b+)}$ | $ALe^{(a-b-)}$ |
| Pylorus | Surface | – | – | – | – | – | – | – |
| | Glands | + + +/– | + + +/– | + + + | + + +/– | + + +/– | + + + | + + +/– |
| Duodenum | Villosities | – | – | – | – | – | – | – |
| | Brunner's glands | + + +/– | + + +/– | + + + | + + +/– | + + +/– | + + + | + + +/– |
| Ileum | Villosities | – | – | – | – | – | – | – |
| | Paneth cells | – | – | – | + + + | + + + | + + + | + + + |
| Right colon | Absorptive cells | –CD | –CD | –C | +C | + + +C | +/–C | +/–C |
| | Goblet cells | – | – | – | +/– | +/– | +/– | + |
| Left colon | | – | – | – | – | – | – | – |

Immunoperoxidase method was performed as previously described (Bara *et al.*, 1988); positive reactions from + to + + + represent increasing intensity; CD = right colon; C = caecum; + + +/– = simultaneous presence of strong (+/– = weak) positive and negative zones on the same tissue sample.

**Figure 2** Photomicrographs of tissue sections stained with MAb FW6 by immunoperoxidase technique; **a**, fetal colonic mucosa (24 week stage), reactive with MAb FW6 ( × 330); **b**, normal left colonic mucosa demonstrating the absence of the FW6 epitope ( × 100); **c**, moderately differentiated colon adenocarcinoma showing predominantly apical cell surfaces ( × 100); **d**, normal gastric mucosa ( × 100), the FW6 epitope is present in the deep corpus glands independent of the individual secretor status, but it is not detected in the foveolar epithelium; **e**, normal terminal ileum, demonstrating the expression of the FW6 epitope in Paneth cells of secretor individuals ( × 436). **f**, normal pancreas ( × 330), the FW6 epitope is present in pancreatic ducts in some cases, acinar cells lack staining in all cases tested.

MAbs had not been available for direct comparative histochemical study, but the flow cytometric pattern of MAb FW6 reaction with myeloid HL-60 cell line does not coincide with one of MAbs FH4 and FH6 tested by Symington *et al.* (1985).

The expression of the FW6 epitope in the Paneth cells of the ileum and the absorptive cells of the right colon seems to be under the control of the secretor gene. This conflicts with the result that the FW6 epitope is found in pylorus glands, in Brunner's glands and on salivary glycoproteins from non-secretors. The expression of the FW6 epitope in the latter cases seems to be dependent on the H-gene coded fucosyltransferase. The secretions of salivary gland on the other hand do not contain H-gene coded fucosyltransferase and, accordingly, allow definitive conclusions on the secretor gene dependency of blood group related epitopes (Oriol *et al.*, 1986). Up to now we do not have an explanation for the secretor-dependent and independent expression of the FW6 epitope found in the different organs tested.

The distribution pattern of the FW6 epitope in the normal

**Table IV** Immunoperoxidase staining of malignant tumours with MAb FW6

| *Malignant tumours** | *Positive cases* |
|---|---|
| Stomach | |
| well differentiated | 2/2[†] |
| moderately differentiated | 1/4 |
| poorly differentiated | 1/2 |
| signet-ring | 1/1 |
| Breast | 0/10 |
| Lung | |
| squamous cell | 0/3 |
| large cell | 2/3 |
| broncho-alveolar | 0/1 |
| mucoepidermoid | 1/1 |
| Pancreas | |
| adenocarcinoma | 0/5 |
| Prostate | |
| adenocarcinomas | 0/4 |
| Ovary | |
| mucinous adenocarcinoma | 2/2 |
| serous carcinoma | 1/2 |
| Testes | |
| seminoma | 0/2 |
| embryonic carcinoma | 0/2 |
| teratocarcinoma | 0/2 |

*Serial paraffin embedded tissue sections; † = the number of positive cases to total number of cases tested is shown.

**Table V** Reactivity of MAb FW6 to various normal tissues and cells

| *Tissue* | *Reactivity* | *Secretor gene dependency* |
|---|---|---|
| Salivary gland | | |
| Serous acini | – | |
| Mucinous tubuli | + + + | independent |
| Stomach | | |
| Foveolar epithelia | – | |
| Gastric glands | + + + | independent |
| Duodenum | | |
| Villi and crypts | – | |
| Brunner's glands | + + | independent |
| Ileum | | |
| Villosities | – | |
| Paneth cells | + + + | dependent |
| Right colon | | |
| Absorptive cells | + | dependent |
| Goblet cells | + | dependent |
| Left colon | | |
| Absorptive cells | – | |
| Goblet cells | – | |
| 24 week stage fetus | | |
| Small intestine | + + | n.d. |
| Colon | + + + | n.d. |
| Kidney | | |
| Glomeruli | – | |
| Henle's loop | – | |
| Urothelia | + | n.d. |
| Lung | | |
| Alveoli | – | |
| Bronchiolar glands | + | n.d. |
| Bronchiolar epithelia | + | n.d. |
| Mammary gland | – | |
| Pancreas | | |
| Interlobular ductuli | + + | n.d. |
| Acinus cells | – | |
| Blood | | |
| Erythrocytes | – | |
| Granulocytes | – | |
| Lymphocyte | – | |
| Bone marrow | – | |
| Testes | – | |
| Ovaries | – | |

n.d. = secretor status not determined.

gastrointestinal tract is also very similar to that published for $Le^y$. $Le^y$-antigen expression for example is very weak or absent in normal colonic epithelium, but is strongly expressed in adenocarcinomas (Brown *et al.*, 1984; Abe *et al.*, 1986; Kim *et al.*, 1986; Sakamoto *et al.*, 1986; Bara *et al.*, 1988). $Le^y$ and extended $Le^y$ structures have also been detected in stomach, duodenum, pancreas and lung (Mollicone *et al.*, 1985; Sun *et al.*, 1987; Kim *et al.*, 1988; Pour *et al.*, 1988; Sakamoto *et al.*, 1989). On the other hand, there are many discrepancies in the distribution of the FW6 epitope and the $Le^y$, extended $Le^y$, or trifucosyl $Le^y$ determinants, which are recognised by several MAbs: 12-4LE (Bara *et al.*, 1988), CC-1, CC-2 (Sun *et al.*, 1987), AH6 (Abe *et al.*, 1983), 75.12 (Blaineau *et al.*, 1983), C14/1/46/10 (Brown *et al.*, 1983), F-3 (Lloyd *et al.*, 1983) and KH-1 (Kaizu *et al.*, 1986). For example, MAbs C14/1/46/10, KH-1, CC-1, CC-2, and AH6 stained acinar cells in half the specimens of normal pancreas and pancreatic adenocarcinomas (Brown *et al.*, 1984; Sun *et al.*, 1987; Kim *et al.*, 1988), whereas FW6 does not. It is questionable why MAb FW6 stains only pancreatic ducts in a few cases. This needs further investigation.

Most important, the staining pattern of MAb 12-4LE does not correspond to the one of MAb FW6 in serial tissue sections of colonic adenocarcinomas. Knowledge concerning the distribution of Y-antigen and its extended derivatives outside the gastrointestinal tract and pancreas is very limited at present. However, in a limited survey by Sun *et al.* (1987) MAbs CC-1 and CC-2 reacted with all breast carcinomas tested, whereas MAb FW6 did not show reactivity at all.

Summarising the results of biochemical epitope analysis and histological epitope distribution of the MAb FW6, there is strong evidence for the detection of a carbohydrate antigen, which is very closely related to the $Le^x/Le^y$-antigen family, without proving identical staining pattern to a known antigen on type 2 chain precursors. The definitive structural characterisation of its carbohydrate epitope is currently under investigation. MAb FW6 might be of probable diagnostic relevance for the detection of colon carcinoma, for the FW6 epitope is demonstrated in sera from colon carcinoma patients.

This work was supported by a grant from the Deutsche Krebshilfe. The authors are grateful to A. Bolte for providing human amniotic fluid. We thank Mrs B. Schöpper and Mrs E. Vierkotten for their skilful technical assistance. MAb CSLEX-1 is a generous gift by Dr Terasaki, Los Angeles, USA, and MAb AM-3 was kindly provided by Dr C. Hanski, Berlin, Germany.

### References

ABE, K., MCKIBBIN, J.M. & HAKOMORI, S. (1983). The monoclonal antibody directed to difucosylated type 2 chain (Fuc α1-2Galβ-1-4(Fucα1-3)GlcNAc; Y Determinant). *J. Biol. Chem.*, **258**, 11793.

ABE, K., HAKOMORI, S. & OHSHIBA, S. (1986). Differentiation expression of difucosyl type chain ($Le^y$) defined by monoclonal antibody AH6 in different locations of colonic epithelia, various histological types of colonic polyps, and adenocarcinomas. *Cancer Res.*, **46**, 2639.

BARA, J., MOLLICONE, R., HERRERO-ZABALETA, E., GAUTIER, R., DAHER, N. & ORIOL, R. (1988). Ectopic expression of the Y ($Le^y$) antigen defined by monoclonal antibody 12-4LE in distal colonic adenocarcinomas. *Int. J. Cancer*, **41**, 683.

BLAINEAU, C., LE PENDU, J., ARNAUD, D., CONNON, F. & AVNER, P. (1983). The glycosidic antigen recognised by a novel monoclonal antibody, 75.12, is developmentally regulated on mouse embryonal carcinomas cells. *EMBO J.*, **2**, 2217.

BROWN, A., FEIZI, T., GOOI, H.C., EMBELTON, J.M., PICARD, J.K. & BALDWIN, K.W. (1983). A monoclonal antibody against human colonic adenoma recognizes difucosylated type-2-blood-group chains. *Biosci. Rep.*, **3**, 163.

BROWN, A., ELLIS, I.O., EMBELTON, M.J., BALDWIN, R.W., TURNER, D.R. & HARDCASTLE, J.D. (1984). Immunohistochemical localization of Y hapten and structurally related H type-2 blood-group antigen on large-bowel tumors and normal adult tissues. *Int. J. Cancer*, **33**, 727.

CORDON-CARDO, C., LLOYD, K.O., FINSTAD, C.L. & 5 others (1986). Immunoanatomic distribution of blood group antigens in the human urinary tract. *Lab. Invest.*, **55**, 444.

FEIZI, T. (1985). Demonstration by monoclonal antibodies that carbohydrate structures of glycoproteins and glycolipids are onco-developmental antigens. *Nature*, **314**, 53.

FUKUSHI, Y., HAKOMORI, S., NUDELMAN, E. & COCHRAN, N. (1984*a*). Novel fucolipids accumulating in human adenocarcinoma. II. Selective isolation of hybridoma antibodies that differentially recognize mono-, di-, and trifucosylated type 2 chain. *J. Biol. Chem.*, **259**, 4681.

FUKUSHI, Y., NUDELMAN, E., HAKOMORI, S. & RAUVALA, H. (1984*b*). Novel fucolipids accumulating in human adenocarcinoma. III. A hybridoma antibody (FH6) defining a human cancer-associated difucoganglioside ($VI^3NeuAcV^3III^3Fuc_2nLc_6$). *J. Biol. Chem.*, **259**, 10511.

FUKUSHIMA, K., HIROTA, M., TERASAKI, P. & 7 others (1984). Characterization of sialosylated Lewis x as a new tumor-associated antigen. *Cancer Res.*, **44**, 5279.

HAKOMORI, S. (1985). Aberrant glycosylation in cancer cell membranes as focused on glycolipids: overview and perspectives. *Cancer Res.*, **45**, 2405.

HANISCH, F.G., EGGE, H., PETER-KATALINIC, J., UHLENBRUCK, G., DIENST, C. & FANGMANN, R. (1985). Primary structures and Lewis blood-group-dependent expression of major sialylated saccharides from mucus glycoproteins of human seminal plasma. *Eur. J. Biochem.*, **152**, 343.

HANISCH, F.G., EGGE, H., PETER-KATALINIC, J. & UHLENBRUCK, G. (1986*a*). Structure of neutral oligosaccharides derived form mucus glycoproteins of human seminal plasma. *Eur. J. Biochem.*, **155**, 239.

HANISCH, F.G., EGGE, H., PETER-KATALINIC, J. & UHLENBRUCK, G. (1986*b*). Primary structure of a major sialyl-saccharide alditol from human amniotic mucins expressing the tumor-associated sialyl-X antigenic determinant. *FEBS Lett.*, **200**, 42.

HANISCH, F.G., UHLENBRUCK, G., PETER-KATALINIC, J. & EGGE, H. (1988). Structural studies on oncofetal carbohydrate antigens (Ca19-9, Ca50, Ca125) carried by O-linked sialyloligosaccharides on human amniotic fluids. *Carbohydr. Res.*, **178**, 29.

HANISCH, F.G., UHLENBRUCK, G., PETER-KATALINIC, J., EGGE, H., DABROWSKI, J. & DABROWSKI, U. (1989). Structures of neural O-linked polyloctasaminoglycans on human skim milk mucins. *J. Biol. Chem.*, **264**, 872.

HANSKI, C., BORNHOEFT, G., TOPF, N., HERMANN, U., STEIN, H. & RIECKEN, E.O. (1990). Detection of a mucin marker for the adenomacarcinoma sequence in human colonic mucosa by monoclonal antibody AM-3. *J. Clin. Pathol.*, **43**, 379.

ITZKOWITZ, S.H., YUAN, M., FUKUSHI, A. & 6 others (1986). Lewis$^x$- and sialylated Lewis$^x$-related antigen expression in human malignant and nonmalignant colonic tissues. *Cancer Res.*, **46**, 2627.

KAIZU, T., LEVERY, S.B., NUDELMAN, E., STENKAMP, R.E. & HAKOMORI, S. (1986). Novel fucolipids of human adenocarcinoma. VI. Monoclonal antibody specific for trifucosyl $Le^y$ ($III^3FucV^3FucVI^2$-$FucnLc_6$), which does not cross-react with $Le^y$ antigen, and a possible conformational epitope structure. *J. Biol. Chem.*, **261**, 11254.

KIM, Y.S., YUAN, M., ITZKOWITZ, S.H. & 5 others (1986). Expression of $Le^y$ and extended $Le^y$ blood group-related antigens in human malignant, premalignant, and nonmalignant colonic tissues. *Cancer Res.*, **46**, 5985.

KIM, Y.S., ITZKOWITZ, S.H., YUAN, M. & 4 others (1988). $Le^x$ and $Le^y$ antigen expression in human pancreatic cancer. *Cancer Res.*, **48**, 475.

KÖHLER, G. & MILSTEIN, C. (1975). Continous culture of fused cells secreting antibody of predefined specifity. *Nature*, **256**, 495.

LAMBOTTE, R. & UHLENBRUCK, G. (1966). Aminomucoids – a new class of hexosamine-rich Glycoproteins. *Nature*, **5059**, 290.

LLOYD, K.O., LARSON, G., STRÖMBERG, N., THURIN, J. & KARLSSON, K. (1983). A mouse monoclonal antibody F-3 recognizes the difucosyl type-2 blood group structure. *Immunogenetics*, **17**, 537.

LLOYD, K.O. (1986). Blood group antigens as markers for normal differentiation and malignant change in human tissues. *Am. J. Clin. Pathol.*, **87**, 129.

MOLLICONE, R., BARA, J., LE PUNDU, J. & ORIEL, R. (1985). Immuno-histologic pattern of type 1 ($Le^a$, $Le^b$) and type 2 (X, Y, H) blood group-related antigens in the human pyloric and duodenal mucosae. *Lab. Invest.*, **53**, 219.

ORIOL, R., LE PENDU, J. & MOLLICONE, R. (1986). Genetics of ABO, H, Lewis, X and related antigens. *Vox Sang.*, **51**, 161.

POUR, P.M., TEMPERO, M.M., TAKASAKI, H. & 4 others (1988). Expression of blood group-related antigens ABH, Lewis A, Lewis B, Lewis X, Lewis Y, and Ca 19-9 in pancreatic cancer cells in comparison with the patient's blood group type. *Cancer Res.*, **48**, 5422.

SAKAMOTO, J., FURUKAWA, K., CORDON-CARDO, C. & 5 others (1986). Expression of Lewis$^a$, Lewis$^x$, X and Y blood group antigens in human colonic tumors and normal tissue and in human tumor-derived cell lines. *Cancer Res.*, **46**, 1553.

SAKAMOTO, J., WATANABE, T., TOKUMARU, T., TAKAGI, H., NAKAZATO, H. & LLOYD, K.O. (1989). Expression of Lewis$^a$, Lewis$^b$, Lewis$^x$, Lewis$^y$, Sialyl-Lewis$^a$, and Sialyl-Lewis$^x$ blood group antigens in human gastric carcinoma and normal gastric tissue. *Cancer Res.*, **49**, 745.

SCHWONZEN, M., KUEHN, N., VETTEN, B., DIEHL, V. & PFREUNDSCHUH, M. (1989). Phenotyping of acute myelomonocytic (AMMOL) and monocytic leukemia (AMOL): association of T-cell-related antigens and skin-infiltration in AMOL. *Leuk. Res.*, **13**, 893.

SOJAR, H.T. & BAHL, O.P.A. (1987). Chemical method for the deglycosylation of proteins. *Arch. Biochem. Biophys.*, **259**, 52.

STÄHLI, C., STAEHELIN, T., MIGGIANO, V., SCHMIDT, J. & HÄRING, P. (1980). High frequencies of antigen-specific hybridomas: dependence on immunization parameters and prediction by spleen cell analysis. *J. Immunol. Meth.*, **32**, 297.

SUN, Q., SIDDIQUI, B., NUDELMAN, E., HAKOMORI, S., HO, J.J.L. & KIM, Y.S. (1987). New murine monoclonal antibodies to a human colonic cancer associated glycolipid, extended difucosylated $Le^y$ glycolipid. *Cancer J.*, **1**, 213.

SYMINGTON, F.W., HEDGES, D.L. & HAKOMORI, S.I. (1985). Glycolipid antigens of human polymorphonuclear neutrophils and the inducible H1-60 myeloid leukemia line. *J. Immunol.*, **134**, 2498.

WOODWARD, M.P., YOUNG, W.W. & BLOODGOOD, R.A. (1985). Detection of monoclonal antibodies specific for carbohydrate epitopes using periodate oxidation. *J. Immunol. Meth.*, **78**, 143.

YUAN, M., ITZKOWITZ, S.H., FERRELL, L.D. & 4 others (1987). Expression of Lewis$^x$ and sialylated Lewis$^x$ antigens in human colorectal polyps. *J. Natl Cancer Inst.*, **78**, 479.